# Enhancers reside in a unique epigenetic environment during early zebrafish development

**DOI:** 10.1186/s13059-016-1013-1

**Published:** 2016-07-05

**Authors:** Lucas J. T. Kaaij, Michal Mokry, Meng Zhou, Michael Musheev, Geert Geeven, Adrien S. J. Melquiond, António M. de Jesus Domingues, Wouter de Laat, Christof Niehrs, Andrew D. Smith, René F. Ketting

**Affiliations:** Institute of Molecular Biology (IMB), Ackermannweg 4, D-55128 Mainz, Germany; Department of Pediatric Gastroenterology, Wilhelmina Children’s Hospital, University Medical Centre Utrecht, 3508 AB Utrecht, The Netherlands; Molecular and Computational Biology, University of Southern California, Los Angeles, CA 90089 USA; Hubrecht Institute-KNAW & University Medical Centre Utrecht, Uppsalalaan 8, 3584 CT Utrecht, The Netherlands; Division of Molecular Embryology, DKFZ-ZMBH Alliance, D-69120 Heidelberg, Germany

**Keywords:** Zebrafish development, Enhancers, DNA methylation, Priming, 4C

## Abstract

**Background:**

Enhancers, not promoters, are the most dynamic in their DNA methylation status throughout development and differentiation. Generally speaking, enhancers that are primed to or actually drive gene expression are characterized by relatively low levels of DNA methylation (hypo-methylation), while inactive enhancers display hyper-methylation of the underlying DNA. The direct functional significance of the DNA methylation state of enhancers is, however, unclear for most loci.

**Results:**

In contrast to conventional epigenetic interactions at enhancers, we find that DNA methylation status and enhancer activity during early zebrafish development display very unusual correlation characteristics: hypo-methylation is a unique feature of primed enhancers whereas active enhancers are generally hyper-methylated. The hypo-methylated enhancers that we identify (hypo-enhancers) are enriched close to important transcription factors that act later in development. Interestingly, hypo-enhancers are de-methylated shortly before the midblastula transition and reside in a unique epigenetic environment. Finally, we demonstrate that hypo-enhancers do become active at later developmental stages and that they are physically associated with the transcriptional start site of target genes, irrespective of target gene activity.

**Conclusions:**

We demonstrate that early development in zebrafish embodies a time window characterized by non-canonical DNA methylation–enhancer relationships, including global DNA hypo-methylation of inactive enhancers and DNA hyper-methylation of active enhancers.

**Electronic supplementary material:**

The online version of this article (doi:10.1186/s13059-016-1013-1) contains supplementary material, which is available to authorized users.

## Background

In recent years, the epigenetic environment in which enhancers reside has been extensively studied. This has been done primarily in cell culture but also in model organisms, including zebrafish, *Xenopus*, mice, and *Drosophila* [1–9]. The results of these studies have led to the identification of two main enhancer types: active and primed. Generally, active enhancers are marked by H3K4me1/2/3 and H3K27ac, have low levels of DNA methylation (hypo-methylated), are bound by transcription factors (TFs), are accessible, and are nucleosome depleted. On the other hand, primed enhancers are enriched for H3K4me1 (and sometimes for H3K27me3), contain more DNA methylation, are less accessible, and are often in the vicinity of key developmental genes (reviewed in [10, 11]).

It is largely unclear what the functional importance is of low DNA methylation levels at active enhancers. It has been documented that a subset of TFs preferentially/exclusively bind to hypo-methylated DNA [12–14]. Interestingly though, a recent study showed that the vast majority of protein DNA interactions are DNA methylation independent [15]. Furthermore, enhancers are enriched for 5-hydroxy-methylcytosine (5hmC), suggesting that the hypo-methylation of enhancers is at least partly an actively regulated process [16–18]. A recent study investigating the role of TET proteins in enhancer biology suggested that the hypo-methylated state of enhancers facilitates fast induction of gene expression upon differentiation [17, 18], implying that the low level of DNA methylation has a direct influence on enhancer functionality. Still, if and how DNA methylation directly influences enhancer activity is not clear and, hence, the functional importance of low DNA methylation levels at active enhancers remains to be clarified.

The mechanisms by which enhancers increase the transcriptional output of genes are also not fully understood. It has been shown that enhancer–promoter contact by looping is essential, but not sufficient, for enhancer function since primed enhancers can be physically associated with target genes prior to their expression [19–24]. Intriguingly, the investigation of individual enhancers revealed that, in some cases, the binding of TFs to the enhancer is essential for looping, whereas in other cases the looping was independent of the presence of the enhancer sequence itself [21, 22].

Recently, the epigenetic landscape during zebrafish development has been studied with a focus on the midblastula transition (MBT), which happens approximately 3.5 h post-fertilization (hpf), and enhancers have also been investigated during later development [6, 25–27]. These studies revealed that H3K4me1 together with H3K27ac mark functionally active enhancers [6]. Interestingly, whereas the mouse and human genomes undergo massive DNA de-methylation after fertilization, the DNA methylation landscape in zebrafish is largely stable post-fertilization [28, 29]. Only very few differentially methylated regions (DMRs) develop between MBT and 8 hpf, whereas prior to MBT and during later development more DMRs were detected [27]. As expected, a subset of the identified DMRs overlapped with enhancers, which is similar to what had been previously described in other organisms [5, 27, 28, 30–32].

In this study we aimed to characterize enhancers during zebrafish early development in more detail by integrating a wide set of different genomic data sets. Surprisingly, we observed that active enhancers are generally hyper-methylated, whereas primed enhancers are mostly hypo-methylated in early zebrafish embryos. This is opposite to what has been described in many other model systems. We found that the inactive, hypo-methylated enhancers (from now on referred to as hypo-enhancers) reside in a unique epigenetic environment, characterized by the co-occurrence of H3K4me1 with H3K4me2/3, and sometimes H3K27me3. Furthermore, they are equally accessible compared with the active enhancers on hyper-methylated DNA (referred to as hyper-enhancers). Our investigation of hypo-enhancers throughout zebrafish development revealed that a subset is actually hyper-methylated in gametes and these specific loci show mild 5hmC enrichment prior to MBT. We also present evidence that hypo-enhancers are already marked with low levels of H3K4me1 prior to MBT, that the H3K4me1 mark is present throughout development, and that a subset is active in a tissue-specific manner later in development. Finally, 4C-Seq analyses of five hypo-enhancers reveals that these are physically associated with the transcriptional start site (TSS) of TF genes and remain stably associated with these genes throughout development, irrespective of the transcriptional status of the gene. In conclusion, we demonstrate that early zebrafish development comprises a time-window in which the “classic” correlations between DNA methylation and enhancer activity do not apply, with DNA hypo-methylation being specific for primed enhancers and DNA hyper-methylation found at active enhancers. These results question a direct relationship between the degree of DNA methylation of these loci and their activity in zebrafish.

## Results

### DNA hyper-methylation associates with enhancer activity during early zebrafish development

**Fig. 1:**
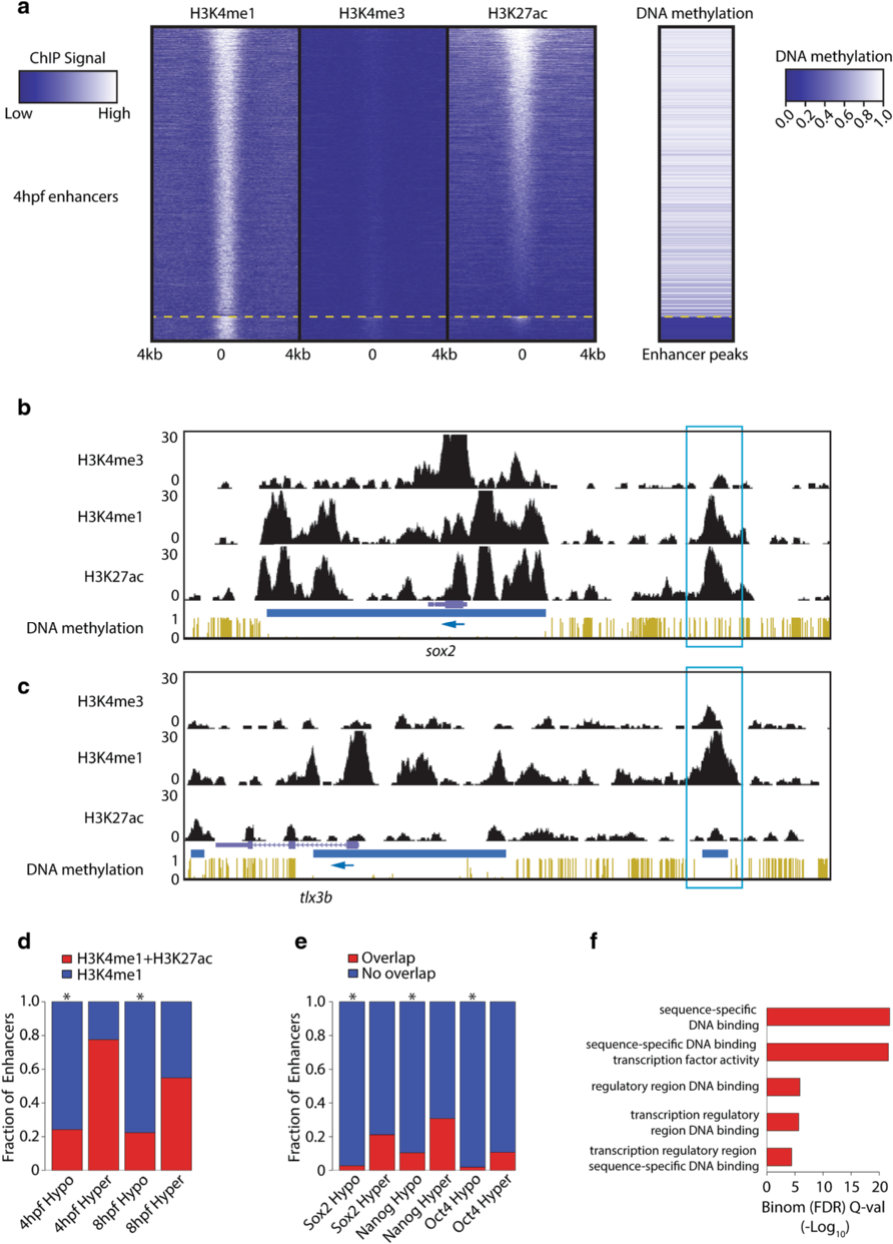
Identification of hypo-enhancers. **a** Heat map displaying H3K27Ac and DNA methylation at enhancers, called by being H3K4me1-positive and H3K4me3-negative at 4 hpf. The heat map was first sorted on DNA methylation, split (*yellow dashed line*), and independently sorted on H3K27ac. **b** Genome browser view of a hyper-enhancer close to the TSS of *sox2*. ChIP-Seq data and DNA methylation data are displayed for embryos 4 hpf. Normalized ChIP-Seq enrichments and fractional methylation are indicated at the *left side* of the image. The *blue box* indicates the hyper-enhancer. The *blue arrow* indicates the orientation of the gene. *Blue horizontal bars* indicate HMRs. **c** Genome browser view of a hypo-enhancer close to the TSS of *tlx3b*. ChIP-Seq data and DNA methylation data are displayed for embryos 4 hpf. Normalized ChIP-Seq enrichments and fractional methylation are indicated at the *left side* of the image. The *blue box* indicates the hypo-enhancer. The *blue arrow* indicates the orientation of the gene. *Blue horizontal bars* indicate HMRs. **d** The overlap of the hypo- and hyper-enhancers with H3K27ac at the indicated developmental time points (*p < 2.2 × 10^−16^, hyper geometric distribution). **e** The overlap of the hypo- and hyper-enhancers with three TFs at 4 hpf (*p < 2.2 × 10^−16^, hyper geometric distribution). **f** Molecular Gene Ontology terms associated with hypo-enhancers using the GREAT tool. Significance score for enrichment is given on the *x-axis. FDR* false discovery rate

The dynamic regulation of DNA methylation at enhancers during development and differentiation prompted us to investigate the relationship between enhancers, DNA methylation, and their dynamics during zebrafish development [32–35]. To this end we generated four different methylomes at 2 hpf (64-cell stage), 4 hpf (dome), 8 hpf (75 % epiboly), and 24 hpf using whole-genome bisulfite sequencing and previously published ChIP-Seq data [6]. We defined putative enhancers as loci with H3K4me1 peaks that do not overlap with an annotated TSS (±4 kb) or an H3K4me3 peak [36]. For simplicity, these putative enhancers are hereafter called enhancers. We further separated those loci into active and inactive enhancers using H3K27ac enrichment as a proxy for enhancer activity [2, 6, 37]. Using previously published ChIP-Seq peaks [6], we identified, depending on the developmental stage, ~10,000–37,000 enhancer loci. To visualize the epigenetic landscape at these enhancers, we analyzed the distribution of multiple epigenetic marks at 4 hpf over enhancers containing at least five CpGs (Fig. 1a). In contrast to what one would expect from previous studies [5, 17, 30, 31], we found that DNA hyper-methylation is associated with the presence of H3K27ac. We performed two additional analyses that show that this non-canonical finding reflects a true biological phenomenon and does not stem from anomalies in either data sets or analysis. First, the data sets do show the canonical association at TSSs between DNA methylation and active histone modifications: low levels of DNA methylation correlate with local enrichment of active histone marks, as shown previously in zebrafish (Additional file 1: Figure S1a) [38]. Second, using publicly available data sets from mouse [39, 40], our own analysis confirms that, indeed, the majority of enhancer peaks tend to be hypo-methylated/partially methylated in mice (Additional file 1: Figure S1b). Importantly, the contrast between the hyper-methylation of the majority of enhancers in zebrafish and the hypo-methylation/partial methylation status of enhancers in mice is not due to general differences between the mouse embryonic stem cell (mESC) and zebrafish methylomes since exons and introns show a similar methylation pattern in mESCs and zebrafish at 4 hpf (Additional file 1: Figure S1c).

To further study global DNA methylation dynamics at active enhancer elements, we identified stage-specific enhancers based on the presence of H3K27ac at specific developmental time points (4 hpf, 8 hpf, and 24 hpf) and probed the DNA methylation status of these loci at these three time points (Additional file 1: Figure S1e). Between 4, 8, and 24 hpf, the average DNA methylation level at these stage-specific active enhancers was largely stable at ~85–90 %, suggesting a minimal effect of enhancer activity on DNA methylation during early zebrafish development.

The global nature of the analyses presented thus far could miss DNA methylation changes at a limited set of specific enhancers. To probe the role of enhancers in shaping the DNA methylation landscape locus-specifically, we checked the amount of DMRs that overlap with an enhancer at 4 or 8 hpf. Again in agreement with recently published data [27], we only found a small number of DMRs (~0.5 % overlap of a DMR at the two time points) that overlap with an enhancer. Importantly, this stability of DNA methylation at enhancers is specific to early development as enhancers that are hyper-methylated during early development can lose DNA methylation in adult muscle (Additional file 1: Figure S1d). In summary, we detected only limited DNA methylation dynamics at active and inactive enhancer elements during early zebrafish development and found that, surprisingly, active enhancers tend to be hyper-methylated during this developmental phase.

### Hypo-enhancers define inactive enhancers close to TFs

The vast majority of enhancers at hypo-methylated loci are not active in any of the embryonic data sets analyzed if we use H3K27ac as a proxy of enhancer activity. To follow up on this observation, we defined two sets of enhancers based on their DNA methylation: hypo-enhancers (<25 % DNA methylation and >5 CpGs, *n* = 774 at 4 hpf) and hyper-enhancers (>75 % DNA methylation and >5 CpGs, *n* = 7722 at 4 hpf). Examples of a hyper- and hypo-enhancers and their epigenetic environments are shown in Fig. 1b and c, respectively. To quantify the fraction of active enhancers among the hypo- and hyper-enhancers, we computed the fraction that overlaps with an H3K27ac peak at 4 and 8 hpf. As shown in Fig. 1a, hypo-enhancers show limited overlap with an H3K27ac peak (*p* < 2.2 × 10^−16^, hypergeometric distribution); only ~24 % of the hypo-enhancers do so (Fig. 1d). We sought to further support these findings by checking TF occupancy of hyper- and hypo-enhancers. For this, we analyzed publicly available ChIP-Seq data sets of three core pluripotency TFs (Nanog, Oct4 and Sox2) [41, 42] and computed the overlap with the two-enhancer types. Consistent with our H3K27ac analysis, hypo-enhancers also have significantly limited overlap with TF binding sites (*p* < 2.2 × 10^−16^, hypergeometric distribution; Fig. 1e).

Finally, we used the GREAT tool [43] to perform Gene Ontology (GO) term analysis to see if hypo- or hyper-enhancers are associated with specific classes of genes. Intriguingly, whereas hyper-enhancers are not associated with genes enriched for a molecular function, hypo-enhancers are only enriched for molecular function GO terms that point towards TFs (Fig. 1f). This shows that the set we marked as hypo-enhancers defines loci that mostly resemble inactive enhancers close to TFs.

### Hypo-enhancers drive reproducible expression in enhancer assays

Previously, Bogdanovic et al. [6] performed a series of enhancer assays revealing that a subset of putative enhancers identified in their study are able to drive expression in a transgenic setting. Overlapping their validated enhancers with our two enhancer classes revealed that all (12/12) enhancers tested by Bogdanovic et al. [6] are hyper-enhancers. This overlap confirms that at least some hyper-enhancers identified in our study drive expression in a transgenic setting. To validate the functionality of the identified hypo-enhancer loci as enhancers, we performed transgenic enhancer assays using the ZED system [44]. We cloned five hypo-enhancers close to genes with a tissue-specific expression pattern, of which four gave reproducible results (Additional file 1: Figure S2a–g). Of these, three resembled the expression pattern of the closest gene (*dacha*, *tbx2a*, and *gsc*). These data show that the identified loci, referred to as hypo-enhancers, are also capable of driving tissue-specific gene expression in transgenic settings. We conclude that at least some hypo-enhancers are enhancers.

### A subset of hypo-enhancers show 5hmC enrichment prior to MBT

**Fig. 2:**
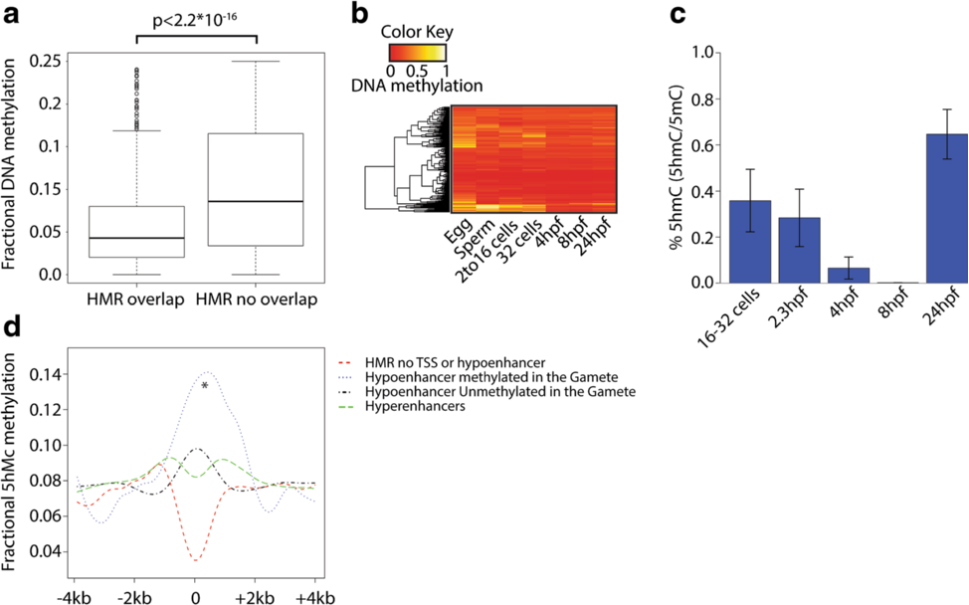
Hypo-enhancers are reprogrammed prior to MBT. **a** The fractional DNA methylation states of all non-TSS-associated HMRs with less than 25 % DNA methylation overlapping and not overlapping a hypo-enhancer. **b** Heat map displaying the DNA methylation status of the hypo-enhancers (defined at 4 hpf) throughout development. **c** Bulk 5hmC quantification using LC-MS/MS. *Error bars* represent the standard deviation derived from biological triplicates. **d** Profile displaying 5hmC distribution within 4 kb of the middle point of all HMRs that do not overlap with a TSS or a hypo-enhancer, stably non-methylated hypo-enhancers, hypo-enhancers that lose DNA methylation during early zebrafish development, and all hyper-enhancers (*p < 2.2 × 10^−16^, non-paired Wilcoxon test)

We compared the DNA methylation status between all non-TSS-associated hypo-methylated regions (HMRs; less than 25 % DNA methylation) that do or do not overlap hypo-enhancers. Strikingly, the HMRs that do overlap hypo-enhancers are significantly less methylated than the HMRs that do not (*p* < 2.2 × 10^−16^, non-paired Wilcoxon-test; Fig. 2a). This could suggest that DNA methylation is specifically suppressed at these enhancer loci. Interestingly, a subset of hypo-enhancers displayed high levels of DNA methylation prior to MBT, particularly in the oocytes (Fig. 2b). This is in line with previous analysis of the same data sets showing that primarily the oocyte methylome is dynamic after fertilization [28, 29]. The oxidation of 5-methylcytosine (5mC) into 5hmC has been proposed as a mechanism to achieve de-methylation [45]. Since immunofluorescence data indicated that 5hmC levels are very low or even absent in zebrafish embryos 3–10 hpf [46], we first checked if 5hmC is present during early zebrafish development by performing liquid chromatography–tandem mass spectrometry (LC-MS/MS) experiments throughout zebrafish development. This analysis revealed low but clearly detectable 5hmC levels (~0.35 % of the methylated cytosines) prior to MBT and levels close to background after MBT (Fig. 2c). The analysis of a TAB-Seq data set generated from 32-cell-stage embryos (1.75 hpf) [29], reflecting hydroxy-methylation (5hmC), revealed significant enrichment of 5hmC over the hypo-enhancer loci that are hyper-methylated in the gametes compared with the other three tested groups (*p* < 2.2 × 10^−16^, non-paired Wilcoxon test). In contrast, the hypo-enhancers that are already hypo-methylated in the gametes did not show such enrichment (Fig. 2d). Given that TET enzymes have been identified as the enzymes that oxidate 5mC to 5hmC [45, 47, 48], our findings would be consistent with a model where TET enzymes target a subset of the hypo-enhancers during early zebrafish development in order to induce the hypo-methylated state of these enhancers; however, this remains to be tested.

### Hypo-enhancers and hyper-enhancers are equally accessible

**Fig. 3:**
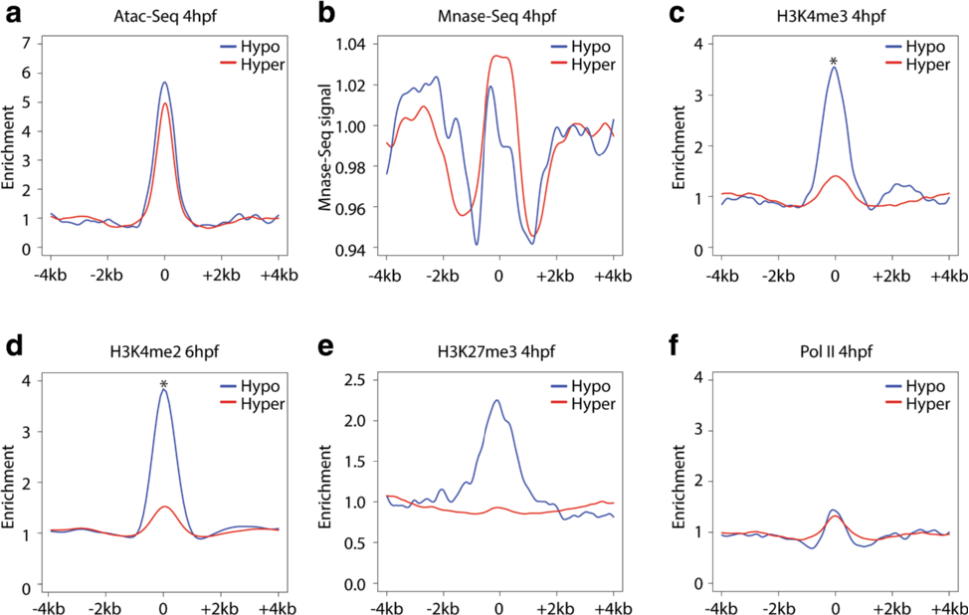
Hypo-enhancers have a unique epigenetic environment. **a**–**f** Profiles of Atac-Seq, Mnase-Seq, H3K4me2/3, H3K27me3, and Pol II ChIP-Seq over hypo- and hyper-enhancers. The enrichment (*y-axis*) of the various epigenetic marks is plotted over a region of 4 kb up- and downstream of the middle points of the two enhancer types

To characterize the physical accessibility of the identified hyper- and hypo-enhancers, we generated an Atac-Seq data set from 4-hpf embryos and made use of an available MNase-Seq data set [49]. We first confirmed that the Atac-Seq and MNase profiles are of high quality by computing their enrichment over TSSs (Additional file 1: Figure S3a). As expected, this revealed nucleosome phasing in both profiles and revealed an inverse correlation between nucleosome occupancy (MNase profile) and accessibility (Atac-Seq). We then probed the accessibility of hypo- and hyper-enhancers. Surprisingly, whereas work in mice and human embryonic stem cells (ESCs) showed that primed enhancers are less accessible and still bound by nucleosomes, both hypo-enhancers and hyper-enhancers are equally accessible in early zebrafish embryos (Fig. 3a, b).

### Hypo-enhancers have a unique epigenetic make up

To further probe epigenetic differences between the two enhancer types, we generated ChIP-Seq profiles for multiple histone modifications at 4 hpf. We started out by computing the enrichment of both H3K4me3 and H3K4me2 over both the hypo- and hyper-enhancers. Unexpectedly, whereas the hyper-enhancers reveal minimal enrichment compared with the background control, the hypo-enhancers reveal significantly stronger enrichment with average enrichment of ~4.5- and ~3-fold for H3K4me2 and H3K4me3, respectively (*p* < 2.2 × 10^−16^, non-paired Wilcoxon test; Fig. 3c, d; Additional file 1: Figure S3b). Furthermore, we showed that the enrichment of H3K4me2/3 over hypo-enhancers is a feature of the majority of hypo-enhancers by plotting all read densities in box plots (Additional file 1: Figure S3c–f). We note that, in both cases, the enrichments are still considerably lower than those observed at TSSs (typically more than tenfold; data not shown). Previous work in ESCs showed that a subset of primed enhancers is enriched for the repressive H3K27me3 mark and this type of enhancer is considered to be poised for activation [37]. We found that only the hypo-enhancers show a small enrichment (Fig. 3e), suggesting that only a few hypo-enhancers are poised for activation. In contrast to other work, we do not find any enrichment of Pol II at either the hypo- or the hyper-enhancers (Fig. 3f) [50].

Finally, we analyzed the epigenetic status of TSSs within 50 kb of either hypo- or hyper-enhancers. This analysis revealed that hyper-enhancers tend to be close to active genes as their proximal TSSs are more enriched for both Pol II and H3K4me3. Furthermore, the hyper-enhancer-associated TSSs are slightly more accessible and nucleosome-poor compared with those associated with hypo-enhancers (Additional file 1: Figure S3g–j). Altogether, these analyses show that hypo- and hyper-enhancers are loci with epigenetically distinct properties in early zebrafish embryos.

### Hypo-enhancers are stable throughout zebrafish development

**Fig. 4:**
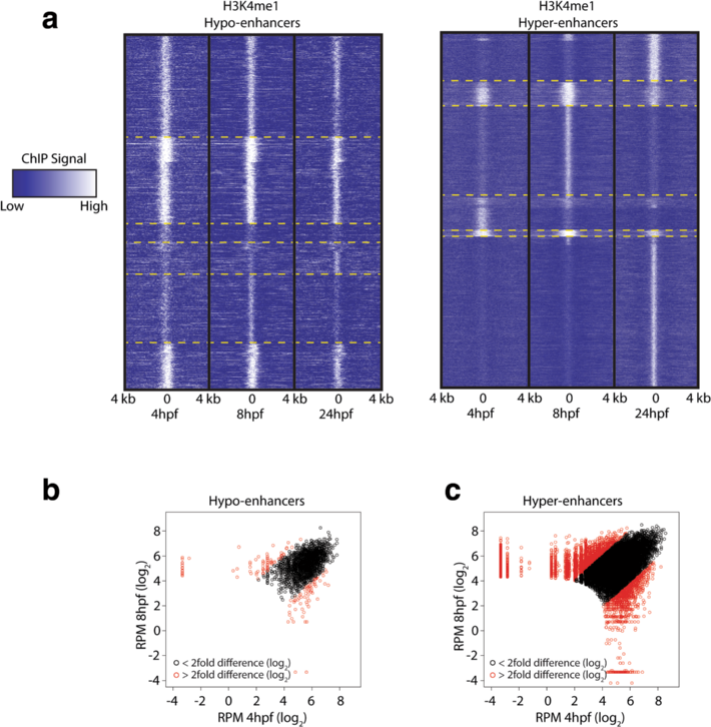
Hypo-enhancers are stable throughout zebrafish development. **a** Heat map displaying RPM-normalized H3K4me1 intensities of all hypo- and hyper-enhancers called throughout the first 24 h of development. The heat map was clustered using k-means clustering (*n* = 6). *Yellow dashed lines* indicate cluster transitions. **b**, **c** Dynamics of H3K4me1-normalized read density of hypo- and hyper-enhancers at 4 and 8 hpf. *Red circles* indicate peaks where the normalized read density changes more than fourfold between the two time points. *Black circles* indicate peaks where the normalized read density changes less than fourfold between the two time points

To address the stability of the H3K4me1 mark at both hypo- and hyper-enhancers, we performed an unbiased, genome-wide analysis of this histone mark throughout the first 24 h of zebrafish development. To do this we called all hypo- and hyper-enhancers throughout development and determined the H3K4me1 enrichment at all time points and visualized this in a heat map (Fig. 4a). This revealed that H3K4me1 at hypo-enhancers is rather stable throughout development and that no clear stage-specific clusters can be observed after k-means clustering. The hyper-enhancers, however, reveal clear stage-specific clusters and the presence of H3K4me1 correlates nicely with enrichment for H3K27ac (Additional file 1: Figure S4). To address the H3K4me1 stability more quantitatively, we computed the normalized read density in an area extending 200 bp on each side of the center of the H3K4me1 peak and compared this between all hypo- and hyper-enhancers called in the 4- and 8-hpf ChIP-Seq data sets (Fig. 4b, c). This analysis revealed largely similar results as observed in the heat map (Fig. 4a): less than 9 % of the hypo-enhancers showed more than a fourfold difference in read density between the two time points, whereas roughly 33 % of the hyper-enhancers show a more than fourfold difference. This shows that the H3K4me1 mark at hypo-enhancers is particularly stable during early zebrafish development, in contrast to hyper-enhancers.

### Hypo-enhancers are marked with H3K4me1 prior to MBT

Although the majority of the histone marks are deposited around MBT, low levels of H3K4me3 at TSSs have been observed prior to MBT [25, 26]. This, in combination with the stable nature of H3K4me1 at hypo-enhancers post MBT, prompted us to investigate the H3K4me1 status of the hypo- and hyper-enhancers prior to MBT by performing H3K4me1 ChIP-Seq at 2.5 hpf (256-cell stage). Initial peak calling revealed a low amount of high-confidence peaks but manual investigation of some of the peaks that were called suggested that these are bona fide H3K4me1 peaks (data not shown). Moreover, an H3K4me1 profile and a heat map generated over all refseq TSSs revealed mild enrichment and the well-known bimodality of this histone modification at TSSs (Additional file 1: Figure S5a, b). These results suggest that the chromatin immunoprecipitation (ChIP) procedure worked appropriately but that the amount of H3K4me1 present at this time point is simply very low, as shown for multiple other histone modifications previously [25, 26]. Indeed, conventional immunofluorescence coupled to confocal microscopy for H3K4me1 on 2-hpf embryos revealed very weak staining, if any, whereas at 4 hpf the presence of H3K4me1 is obvious (Additional file 1: Figure S5c).

We also generated H3K4me1 profiles over the identified hypo- and hyper-enhancers (called from 4-hpf embryos). In line with the stability of this mark at hypo-enhancers later in development, the hypo-enhancers display a clear, ~2.5-fold enrichment of H3K4me1 at 2.5 hpf (Additional file 1: Figure S5d). This enrichment is significantly higher than that observed over the hyper-enhancers (*p* < 2.2 × 10^−16^, non-paired Wilcoxon test). Furthermore, representation of these data in box plots reveals this is a global trend that includes a large fraction of the hypo-enhancers (Additional file 1: Figure S5e, f). Together, these data show that some hypo-enhancers are pre-marked by H3K4me1 at 2.5 hpf.

### Hypo-enhancers overlap with H3K27ac peaks in adult zebrafish

**Fig. 5:**
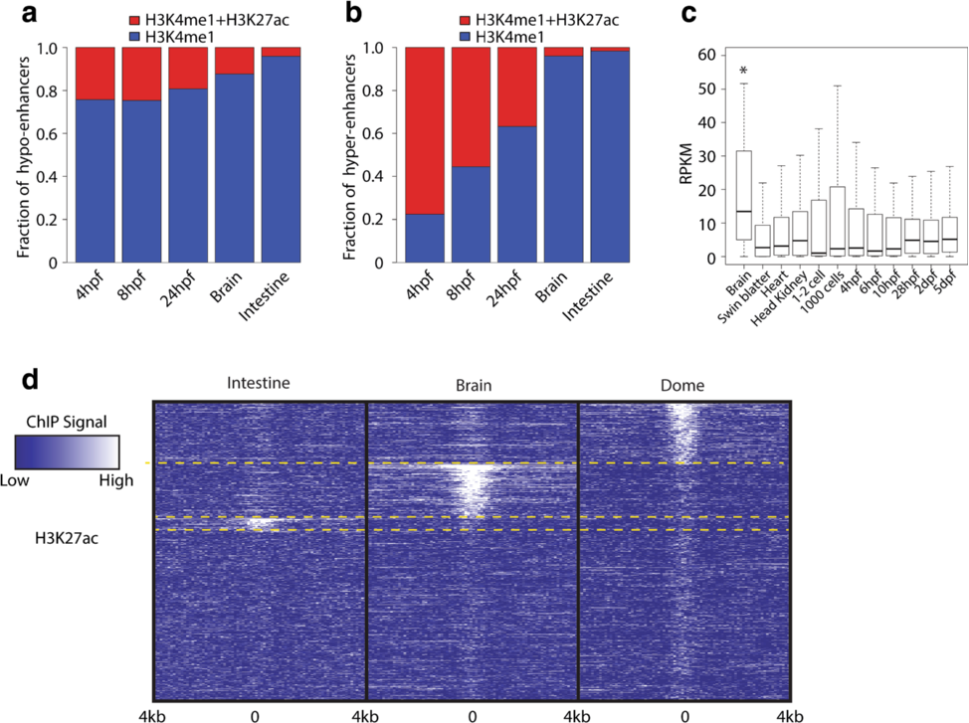
Hypo-enhancers are active in adult tissues. **a**, **b** The fraction of hypo-enhancers (**a**) and hyper-enhancers (**b**) overlapping with a H3K27ac peak at the indicated developmental stages and tissues. **c** Box plot displaying expression values (RPKM) of genes within 20 kb of a hypo-enhancer overlapping with H3K27ac in brain. Expression data were obtained from publicly available RNA-Seq data from different tissues and developmental stages (indicated on the *x-axis*. *p < 0.05, paired Wilcoxon test, brain compared to all other samples. **d** Heat maps of normalized H3K27ac signal over hypo-enhancers in two different tissues (*Intestine* and *Brain*) and at 4 hpf (*Dome*). The heat map was sorted on brain, split, and subsequently sorted on intestine, etc. *Yellow dashed lines* indicate sorting transitions

In our data sets derived from early embryos, the majority of hypo-enhancers (~80 %) never overlap with a high confidence H3K27ac peak (Fig. 5a), while most hyper-enhancers do (Fig. 5b). Furthermore, all five enhancers tested in enhancer assays did not show any activity before 8 hpf. This raises the question of whether hypo-enhancers perhaps become active later in development or even only in the adult. To address this, we generated two H3K27ac ChIP-Seq data sets from two adult tissues (intestine and brain). This revealed that ~4 and ~12 % of the hypo-enhancers called at 4 hpf overlap with H3K27ac peaks found in the intestine and brain, respectively (Fig. 5a, d). The majority of hypo-enhancers that are H3K27ac-positive in the two adult tissues are H3K27ac-negative at 4 hpf and vice versa (Fig. 5d). The enhancers called in brain could be probed for activity using publicly available RNA-Seq data from multiple adult tissues. After linking the hypo-enhancers that are active in brain to their closest TSS (within 50 kb), the expression of the associated refseq genes was plotted throughout development and in four different tissues. This analysis revealed that H3K27ac-marked hypo-enhancers in the adult brain are in the proximity of genes that show a significant increase in expression in the brain compared with all other RNA-Seq data sets tested (*p* < 0.05, paired Wilcoxon test; Fig. 5c). Together, these data show that hypo-enhancers, defined early in embryonic development, are tissue-specific enhancers that strongly correlate with gene expression in adult tissues.

### Hypo-enhancers associate with TSSs of target genes independent of their transcriptional activity

**Fig. 6:**
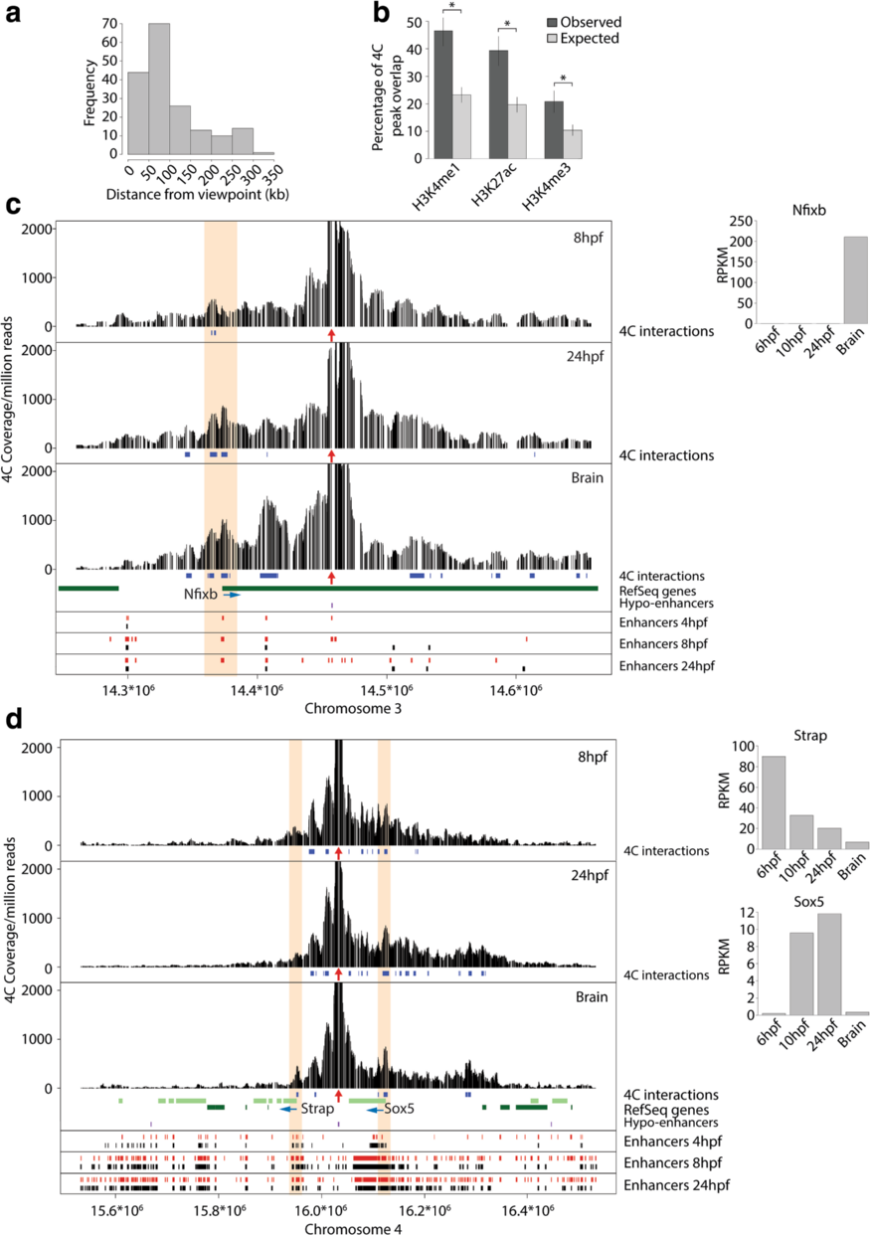
Hypo-enhancers interact with target gene promoters throughout development. **a** Distribution of distances from viewpoints of all non-redundant 4C interactions observed. The five viewpoints analyzed by 4C-Seq were pooled. **b** Overlap between all non-redundant 4C interaction sites and ChIP-Seq peaks observed and expected by chance as indicated (*p < 2.2 × 10^−16^, non-paired Wilcoxon-test). **c**, **d** 4C-Seq profiles generated at 8 and 24 hpf and from brain. *Red vertical arrows* under every 4C-Seq panel indicate the viewpoint. All significant 4C interactions are indicated by *blue rectangles* below every 4C-Seq panel and 4C interactions with a TSS are highlighted in *orange*. Below the 4C-Seq panels, tracks indicating hypo-enhancers (*purple*) and ChIP-Seq peaks (H3K4me1, *red*; H3K27ac, *black*) throughout development are shown. To the *right* of the 4C-Seq panels we depict the expression of the indicated genes at the indicated developmental time points or tissue

The above-described stability of the H3K4me1 mark at hypo-enhancers throughout development and their late activation in adult tissues suggested that the hypo-enhancers are primed for activation later in development. These observations prompted us to determine whether the three-dimensional architecture of the hypo-enhancers is also in a primed configuration throughout development and in adult brain. To do this we generated 4C-Seq data from 8- and 24-hpf embryos and adult brain for five different hypo-enhancers with different epigenetic signatures. After mapping and processing of the sequenced reads, we used a peak-calling algorithm to identify 4C interactions. Initial analysis revealed that the majority of 4C interactions are located within 100 kb of the viewpoint, although some interactions were up to 350 kb away (Fig. 6a). Furthermore, computing the overlap of non-redundant 4C interactions from all embryonic 4C experiments with H3K4me1-, H3K27ac-, and H3K4me3-enriched loci throughout development showed that ~46 ~39, and ~20 % of the 4C interactions overlap with H3K4me1, H3K27ac, and H3K4me3, respectively (Fig. 6b). This shows that the analyzed hypo-enhancers are enriched to interact with functional genomic elements.

Next, we analyzed hypo-enhancer–promoter interactions specifically. As expected for enhancers, for all five tested hypo-enhancers we found one or more 4C interactions close to a promoter. Importantly, we found that the hypo-enhancer interacts with the promoter of a TF in all five cases (Fig. 6c, d; Additional file 1: Figure S6), consistent with the observed GO term enrichment of TFs close to hypo-enhancers (Fig. 1f).

Because we performed the 4C-Seq experiments at two developmental time points and in adult brain, we could also assess the dynamics of the enhancer–promoter interactions throughout development and in adult brain. Focusing only on the hypo-enhancer interactions with a TSS of a TF gene, we detected promoter–enhancer interaction at all three time points in five out of five cases (Fig. 6c, d; Additional file 1: Figure S6).

As described previously [22, 23, 51], we could confirm that promoter–enhancer interactions are already present prior to gene expression (defined as RPKM values <1 in RNA-Seq experiments) in three out of five hypo-enhancer interactions with a TF promoter at 8 hpf (Fig. 6c; Additional file 1: Figure S6b, c). Surprisingly, *sox5* and *meis2a* are not expressed significantly in the brain (RPKM <1) but are still physically associated with a hypo-enhancer in this tissue (Fig. 6d; Additional file 1: Figure S6c). In summary, we show that all tested hypo-enhancers physically associate with the promoter of a TF. These enhancer–promoter interactions are already observed prior to transcriptional activation and are maintained in adult tissue, irrespective of the transcriptional activity of the target gene.

## Discussion

The epigenetic landscape in which enhancers reside has received a lot of attention in recent years. In this study we integrate a wide range of epigenetic data sets and show that active distal regulatory elements are generally hyper-methylated (hyper-enhancers) early in zebrafish development, whereas inactive enhancers are enriched at hypo-methylated DNA (hypo-enhancers). These results are highly unexpected since mammalian systems display opposite global enhancer–DNA methylation correlations [17, 31, 32]. Furthermore, we show that the epigenetic make-up is strikingly different for the hyper- and hypo-enhancers . Even though primed enhancers can be hypo-methylated in human ESCs, primed states generally correlate with hyper-methylation, while active states generally correlate with hypo-methylation [17, 31, 32, 52]. Hence, the opposite correlations we find during early zebrafish development are surprising and represent a unique architecture of enhancers.

### Enhancer activity does not influence DNA methylation

We describe that enhancer activity during zebrafish embryogenesis has minimal effect on the underlying DNA methylation state and vice versa. These findings are striking since there is compelling evidence that low levels of DNA methylation at enhancers do have functional importance in mammalian systems [17, 18]. Interestingly, the inverse association between DNA methylation and enhancer activity seems similar to promoter methylation during early *Xenopus* embryogenesis and mouse spermatogenesis, where some genes that are highly expressed are nevertheless hyper-methylated at the TSSs [53, 54]. These observations show that a temporal uncoupling of DNA methylation and transcriptional repression can take place. The question remains why active enhancers remain hyper-methylated during early zebrafish development. We currently cannot provide a good answer to this question, but it is of great importance to know that DNA methylation dynamics at enhancers in zebrafish can follow different rules to those described thus far in mammalian systems. Mechanistically, insufficient TET activity could be the basis for the lack of de-methylation of active enhancers, as studies on mESCs have shown a direct role for TET enzymes in enhancer demethylation [17, 18]. TET activity is more pronounced in adult zebrafish tissues and, indeed, active enhancers are hypo-methylated in adult tissues [46]. Still, the functional relevance of these differences in DNA methylation behavior between developmental stages remains unresolved.

### Hypo-methylation as a potential driver for enhancer priming

The activation of the zygotic genome and the priming of inactive enhancers are crucial for early development. In *Drosophila*, a single key factor, Zelda, has been identified to bind both active and inactive enhancers and the association of Zelda with inactive genes is crucial for their future activity [55, 56]. However, Zelda is not conserved in vertebrates. We hypothesize that the hypo-enhancers we have defined are in fact primed due to their hypo-methylated state. Interestingly, a similar mechanism has been described for non-methylated CpG islands that gain H3K4me3 due to their low levels of DNA methylation irrespective of the genomic context [57]. Moreover, a study in mESCs lacking Lsh, a factor described to be required for DNA methylation at specific loci, has shown that locus-specific loss of DNA methylation coincides with a gain in H3K4me1 specifically at those regions [58], showing that low DNA methylation can trigger de novo acquisition of H3K4me1. The identification and characterization of the factors that set the H3K4me1 mark at hypo-enhancers in zebrafish will be of pivotal importance in addressing the hypothesis that enhancer priming is initiated by DNA hypo-methylation.

### H3K4me1 is present at hypo-enhancers prior to MBT

In zebrafish, the majority of histone modifications are largely absent prior to MBT, although low levels of H3K4me3,for instance, have been described at these early stages [25, 26]. In this study we present evidence that hypo-enhancers bear low levels of H3K4me1 prior to MBT (at ~2.5 hpf). The significance of these low levels of H3K4me1 at primed hypo-methylated enhancers is currently unclear but they suggest that the marking of primed enhancers is not coupled to transcription since zygotic transcription of the nuclear genome is minimal at this stage [59]. Furthermore, this limited zygotic transcription also implies that parentally inherited factors are responsible for the earliest priming of enhancers. Additional studies are needed to identify the factor(s) responsible for the positioning of H3K4me1 at hypo-methylated enhancers prior to zygotic gene activation.

### Hypo-enhancers are stably associated with TF promoters

The mechanism(s) behind enhancer–promoter looping are poorly understood but multiple factors have been shown to play a role in the formation of enhancer loops, including cohesin, CTCF, and also TFs themselves [19, 20]. Our results demonstrate that hypo-enhancers can associate with promoters long before they become active, when they have epigenetic signatures that mark them as primed enhancers. Perhaps more surprisingly, we also found that these enhancers can stay associated with a target promoter long after the gene has been shut down. This situation has also been observed for the *HoxA* and *HoxD* loci previously [60, 61]. The mechanism behind this observation remains to be investigated but, in this light, it is interesting that the enhancer region of the SHH (sonic hedgehog) gene still contacts the SHH gene in the physical absence of the enhancer itself [22]. This raises the interesting possibility that hypo-enhancers may interact with target promoters through enhancer-independent mechanisms.

## Conclusion

During early zebrafish development, enhancer activity generally has an inverse association with DNA methylation. Furthermore, hypo-methylated enhancers identified early in zebrafish development are active in adult zebrafish and physically associated with target promoters irrespective of their expression.

## Methods

### Bisulfite sequencing library preparations

Total genomic DNA was extracted from carefully timed embryos at indicated developmental stages (2, 4, 8, and 24 hpf). Bisulfite sequencing (BS-Seq) libraries were prepared as described before [30].

### BS-Seq analysis

Reads were mapped to the zebrafish genome assembly (Zv9). DMR and HMR calling was done as described elsewhere [62]. DMRs were filtered by asking for at least five significant different CpGs (*p* < 0.05, Fisher exact test). Additional BS-Seq data sets were downloaded from Potok et al. [28], Jiang et al. [29], Stadler et al. [31], and Wang et al. [39]. In the case where the 4-hpf hypo-enhancers were followed throughout development, only those hypo-enhancers that had more than an average fourfold coverage in all methylomes are displayed and further analyzed. Hypo-enhancers with 0.25 fractional DNA methylation more in the gametes than at 4 hpf were considered methylated in the gametes.

### ChIP-Seq and Atac-Seq analysis

ChIP-Seq was performed essentially as described by Bogdanovic et al. [6] and Atac-Seq was performed as described by Buenrostro et al. [63]. The following antibodies were used in this study: H3K4me1 ChIP grade (abcam), H3K27ac ChIP grade (abcam), and H3K4me2 ChIP grade (abcam). For the 2.5-hpf time point ~8000 embryos were used. The ChIP-Seq libraries were prepared using the Ovation Ultralow Library System V2 Workflow (NuGEN). In brief, the DNA ends were repaired, adapter ligated, and PCR amplified. Size selection of libraries between 250 and 500 bp was done on a LabChip XT instrument (PerkinElmer). These size-selected fractions were pooled and sequenced on a HiSeq 2500 (Illumina). Additional data sets were downloaded from Bogdanovic et al. [6] and Zhang et al. [49]. Peaks of Oct4, Sox2, and Nanog were downloaded from Leichsenring et al. [41] and Xu et al. [42]. For the initial enhancers, identification peaks called by Bogdanovic et al. were used. Enhancers were defined as a H3K4me1 peaks not overlapping a H3K4me3 peak or a RefSeq (zv9) or Ensemble (zv9) annotated TSS (±4 kb). For all other data sets used reads were mapped to the zebrafish genome assembly (Zv9) using Bowtie (v0.12.08) using the following settings: -m10 -n2 -l28 --best --strata --chunkmbs 256. In the subsequent analysis multiple reads mapping to the same location and strand have been collapsed to single reads and only uniquely placed reads were used for peak-calling. The Cisgenome v.2 software package was used for peak-calling (false discovery rate <0.05). A combination of custom PERL, Python, R scripts, BedTools, and Cisgenome functions were used for computational data analysis. In brief, all heat maps and profiles presented represent normalized (RPM) read densities and the background was set to 1 in the case of the profiles. HMRs not overlapping a TSS were defined as being more the 4 kb away from a TSS, similar to enhancers. To compute the stability of H3K4me1 throughout development over hypo- and hyper-enhancers, we computed the average read density ±200 bp from the mid-point of the enhancer. We only considered enhancers with >20 RPM combined over the three time points, and in the case where a time point had an average of 0 RPM, a 0.1 pseudo count was added to facilitate LOG transformation. In cases where the epigenetic status of TSSs close to hypo- and hyper-enhancers was evaluated, we only considered TSSs within 50 kb of a hypo- or hyper-enhancer. The Pol II density over TSSs was calculated as average RPM values in 100-bp bins within ±500 bp of the TSS.

### 4C-Seq

4C-Seq was essentially performed as described in van de Werken et al. [64]. In brief, single cell suspensions were made from the zebrafish embryos and brain using TrypLE (Life Technologies). Subsequently, the usual 4C protocol was followed using DPNII as the primary cutter and Csp6I as the secondary cutter. 4C template DNA from multiple (at least three) independent 4C experiments was pooled and subsequently 0.8–2 μg of template DNA was used in the 4C PCR reaction. 4C libraries were sequenced on the illumina HiSeq platform. The sequence of the primers used in the 4C-seq experiments can be found in Additional file 2.

### 4C-Seq data analysis

For peak-calling in a single 4C experiment, we performed explicit background modeling of the up- and downstream genomic regions. We assumed that, in a completely unstructured chromatin fiber, the contact probability monotonically decreases as a function of the distance to the viewpoint. We modeled this by performing monotonic regression of the 4C signal as a function of the distance to the viewpoint. For this we used the R package isotone, which implements the monotonic regression. We then compared the observed 4C signal to the predicted value from the background model and called the extremes that reach a significance threshold as peaks. For a given threshold q and a distribution F of residuals from the background model, every observation greater than Q3(F) + q × IQR(F), where Q3 is the third quartile of F and IQR(F) the inter-quartile range, is considered significant.

Parameters were as follows for calling a 4C interaction: q between 1.5 and 4 and minimal distance to viewpoint >20 kb.

For computing the overlap between 4C interactions and ChIP-Seq peaks, a non-redundant list of 4C interactions (while merging 4C interactions within 1 kb of each other) was intersected with non-redundant ChIP-Seq lists as indicated. The background overlap was computed by generating semi-random 4C peaks within 500 kb of the originally called 4C peak. These semi-random 4C peaks were thereafter intersected with the non-redundant ChIP-Seq list. The standard deviation for the observed and expected overlap was computed by bootstrapping 10,000 times.

### RNA-Seq analysis

Data sets were downloaded from Collins et al. [65] and Pauli et al. [66]. Reads were mapped to zebrafish genome assembly version danRer7 (Zv9) by tools in the RMAP package [67]. Gene expression was estimated by mapping reads to the transcriptome and computing RPKM for each refseq gene. Here we mapped the two ends of pair-end data separately, but if both ends of one pair were mapped to the same exon, only one was counted in the RPKM. The mappable length of one gene was adjusted based on mapping deadzones.

### Enhancer assays

Enhancer assays were essentially performed as described by Bessa et al. [44]. In the case of the Gsc and Tbx2a enhancer, the activity was scored in primary injected embryos. In the case of Gsc the specific expression pattern was observed in four out of seven transgenic embryos and the Tbx2a enhancers were observed in three out of five embryos. Dacha and Unxc4*.1* enhancer activity was assayed in a transgenic assay. In total two out of two (Dacha), and two out of two (Unxc4*.1*) transgenic founders showed the presented expression pattern. The sequence of the primers used to clone the enhancer elements can be found in Additional file 3.

### LC-MS/MS analysis

DNA was purified from staged embryos by phenol chloroform extraction and subsequently digested to nucleosides using nuclease P1 (Roche), snake venom phosphodiesterase (Worthington), and alkaline phosphatase (Fermentas) and subjected to stable isotope dilution LC-MS/MS analysis as described in detail elsewhere [68].

## Additional files

Additional file 1: Figures S1–S6. (ZIP 3593 kb) Additional file 2: An excel file listing the primers used in the 4C-Seq analysis. (XLSX 41 kb) Additional file 3: An excel file listing the primers used to clone the enhancers in the transgenic assays. (XLSX 47 kb)
