# Supplementary material for: Enhancers reside in a unique epigenetic environment during early zebrafish development
Source: Genome Biol. 2016 Jul 5;17:146. doi: 10.1186/s13059-016-1013-1 (PMC4934011; doi:10.1186/s13059-016-1013-1)
Supplement: Additional file 1: — Figures S1–S6. (ZIP 3593 kb) [file 13059_2016_1013_MOESM1_ESM.zip › Supplemental Figures.pdf]

Figure S1

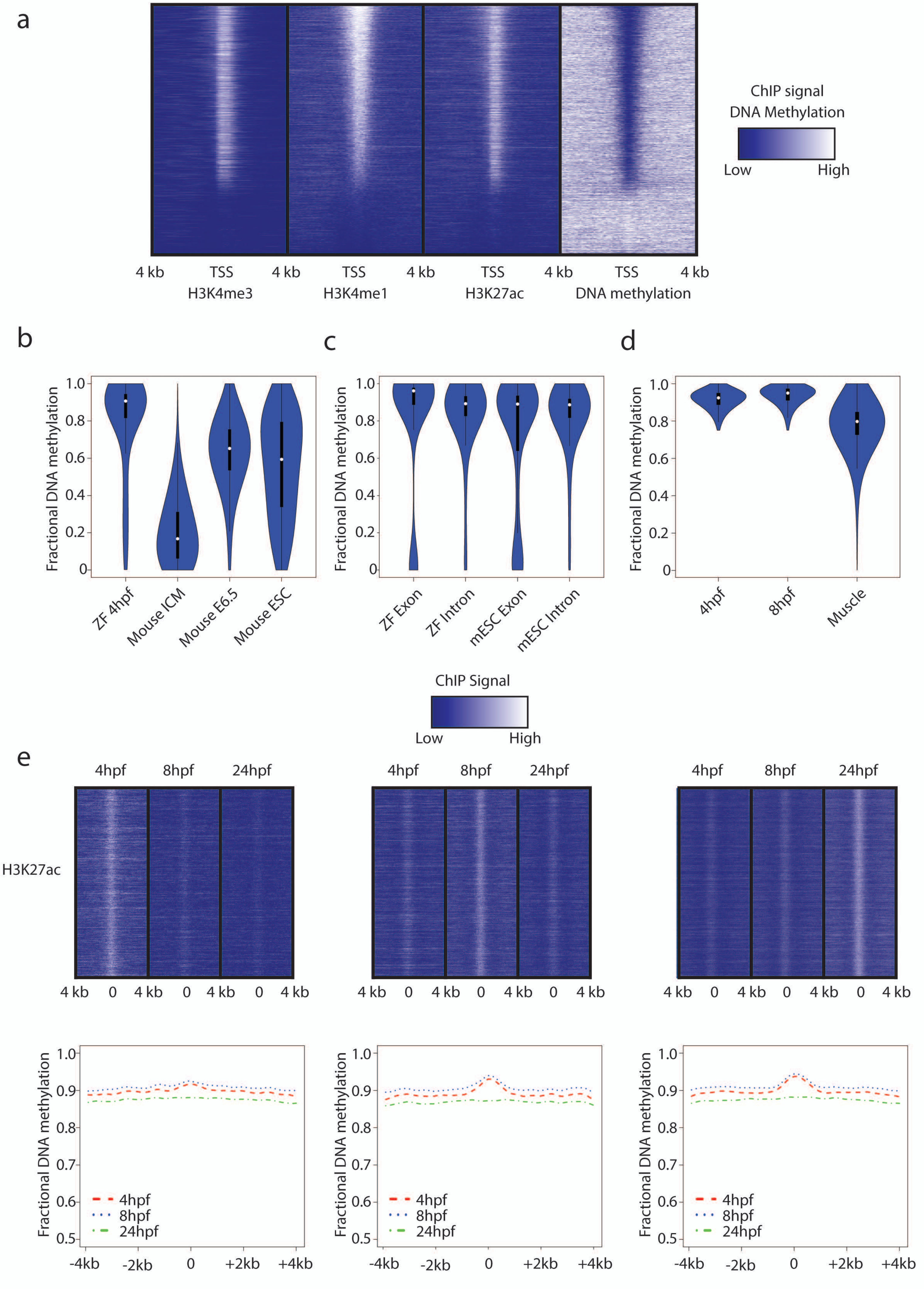
a
TSSs at 4hpf
ChIP signal
DNA Methylation
Low
High
4 kb
TSS
4 kb
TSS
4 kb
TSS
4 kb
TSS
4 kb
H3K4me3
H3K4me1
H3K27ac
DNA methylation
b
c
d
Fractional DNA methylation
1.0
0.8
0.6
0.4
0.2
0
ZF 4hpf
Mouse ICM
Mouse E6.5
Mouse ESC
ZF Exon
ZF Intron
mESC Exon
mESC Intron
4hpf
8hpf
Muscle
ChIP Signal
Low
High
e
4hpf
8hpf
24hpf
H3K27ac
4 kb
0
4 kb
0
4 kb
0
4 kb
Fractional DNA methylation
1.0
0.9
0.8
0.7
0.6
0.5
4hpf
8hpf
24hpf
-4kb
-2kb
0
+2kb
+4kb

# Figure S2

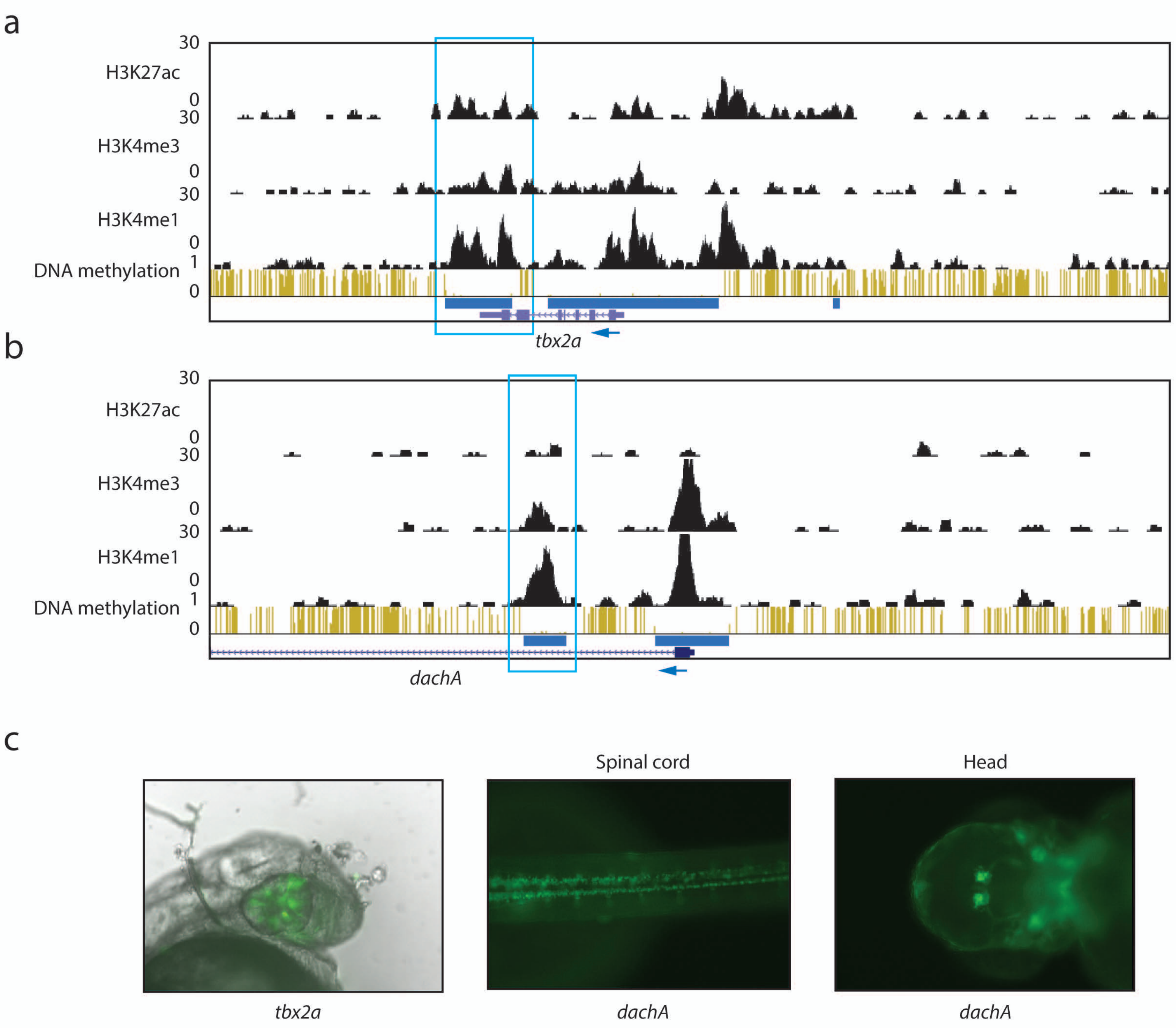
a
30
H3K27ac
0
30
H3K4me3
0
30
H3K4me1
0
1
DNA methylation
0
tbx2a
b
30
H3K27ac
0
30
H3K4me3
0
30
H3K4me1
0
1
DNA methylation
0
dachA
c
Spinal cord
Head
tbx2a
dachA
dachA

# Figure S2

d

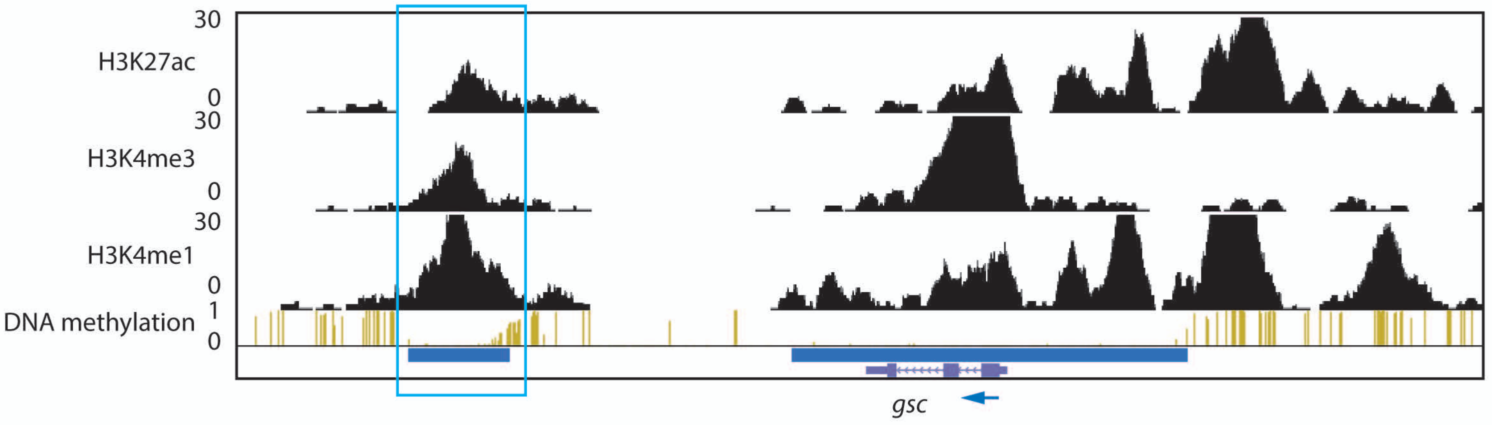


e

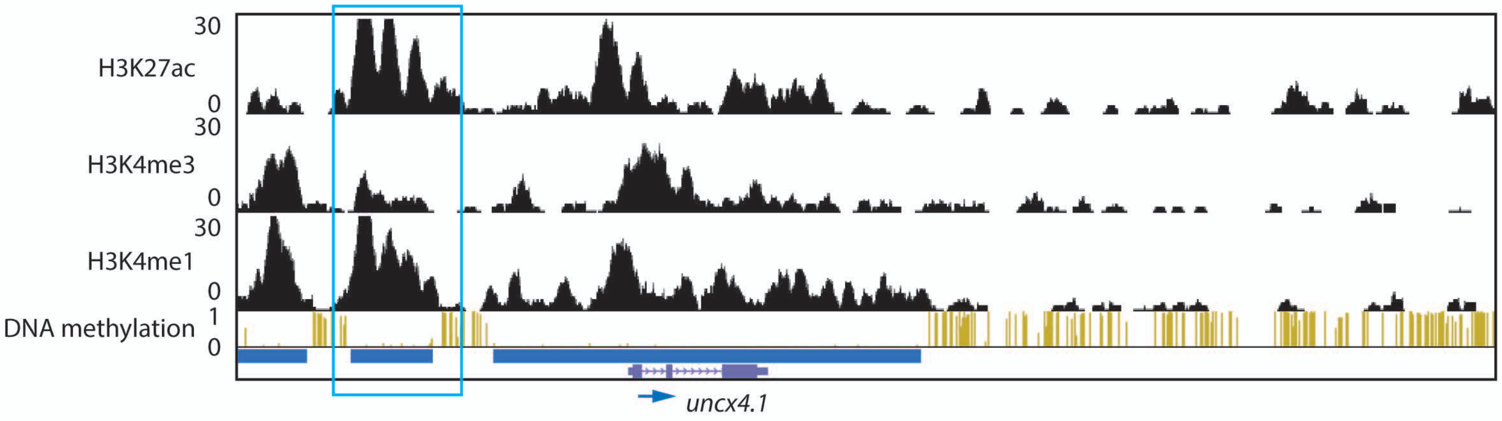


f

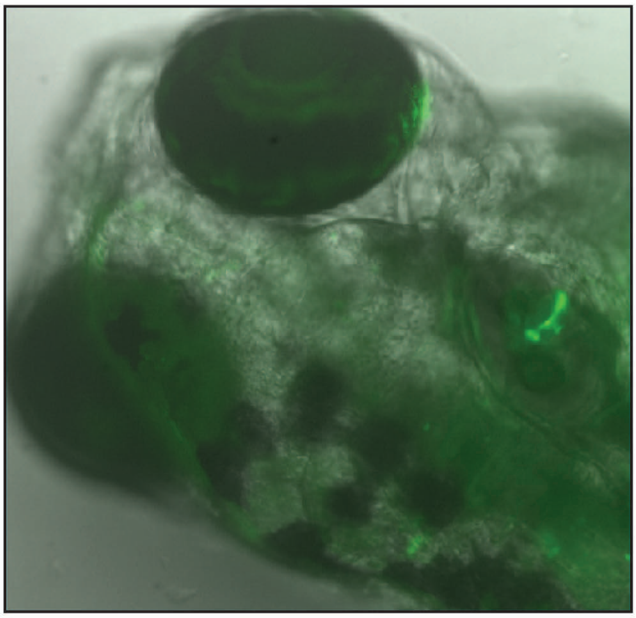

*gsc*

g

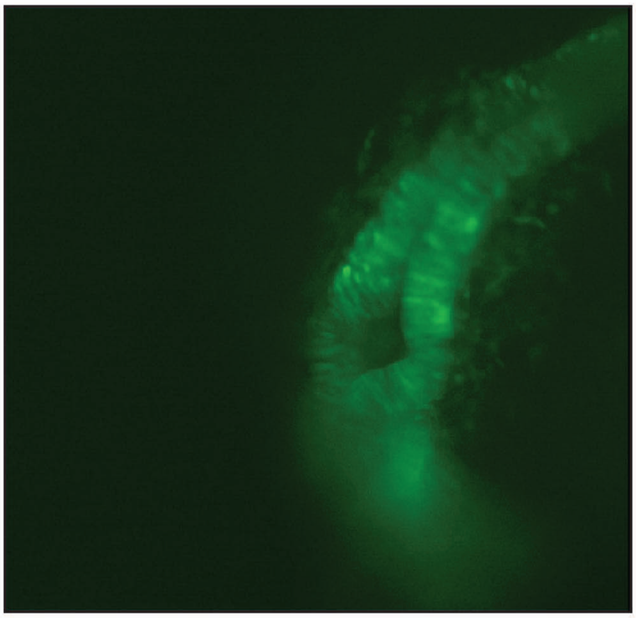

*uncx4.1*

# Figure S3

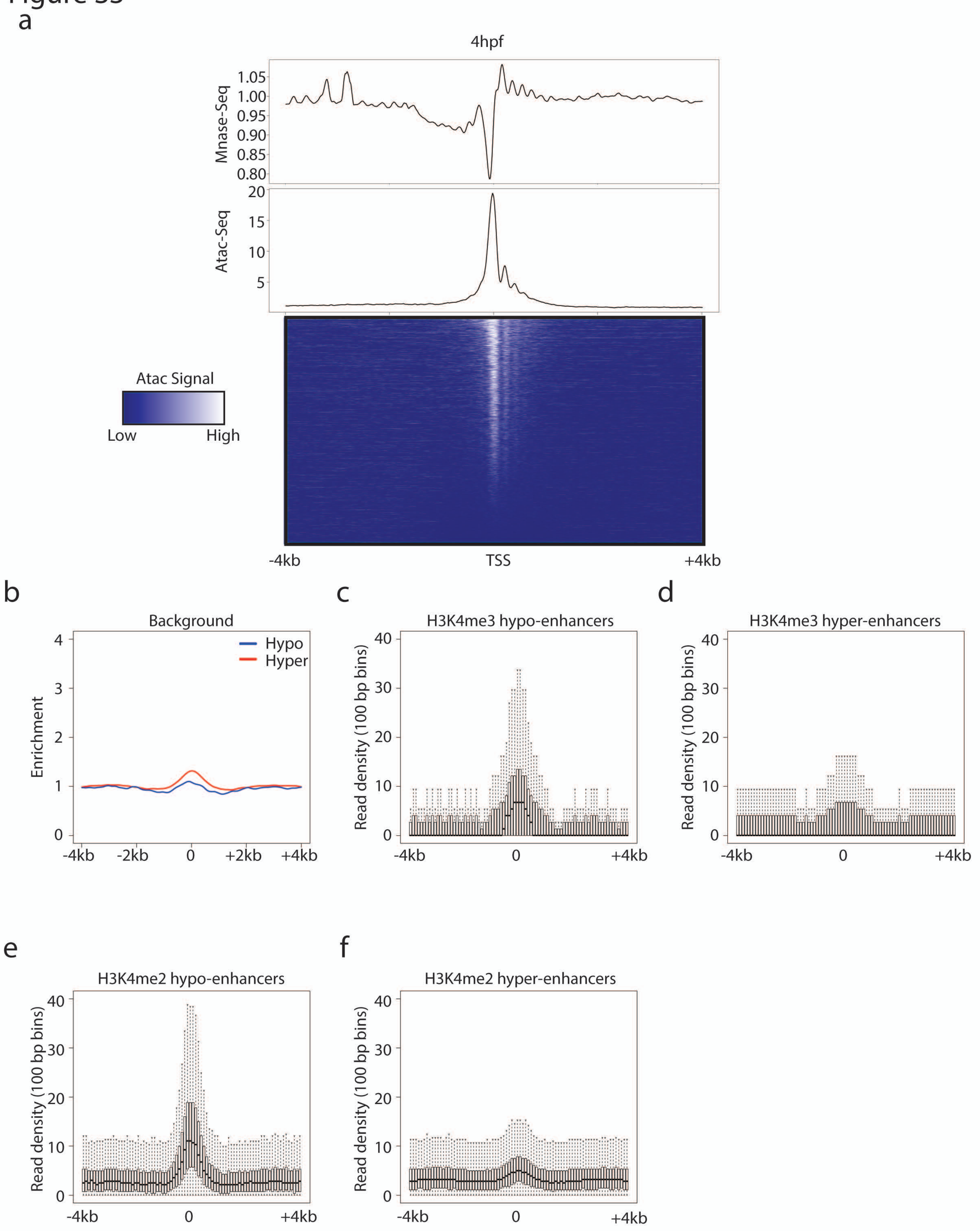
a
4hpf
Mnase-Seq
1.05
1.00
0.95
0.90
0.85
0.80
Atac-Seq
20
15
10
5
Atac Signal
Low
High
-4kb
TSS
+4kb
b
Background
Hypo
Hyper
Enrichment
4
3
2
1
0
-4kb
-2kb
0
+2kb
+4kb
c
H3K4me3 hypo-enhancers
Read density (100 bp bins)
40
30
20
10
0
-4kb
0
+4kb
d
H3K4me3 hyper-enhancers
Read density (100 bp bins)
-4kb
0
+4kb
e
H3K4me2 hypo-enhancers
Read density (100 bp bins)
-4kb
0
+4kb
f
H3K4me2 hyper-enhancers
Read density (100 bp bins)
-4kb
0
+4kb

Figure S3

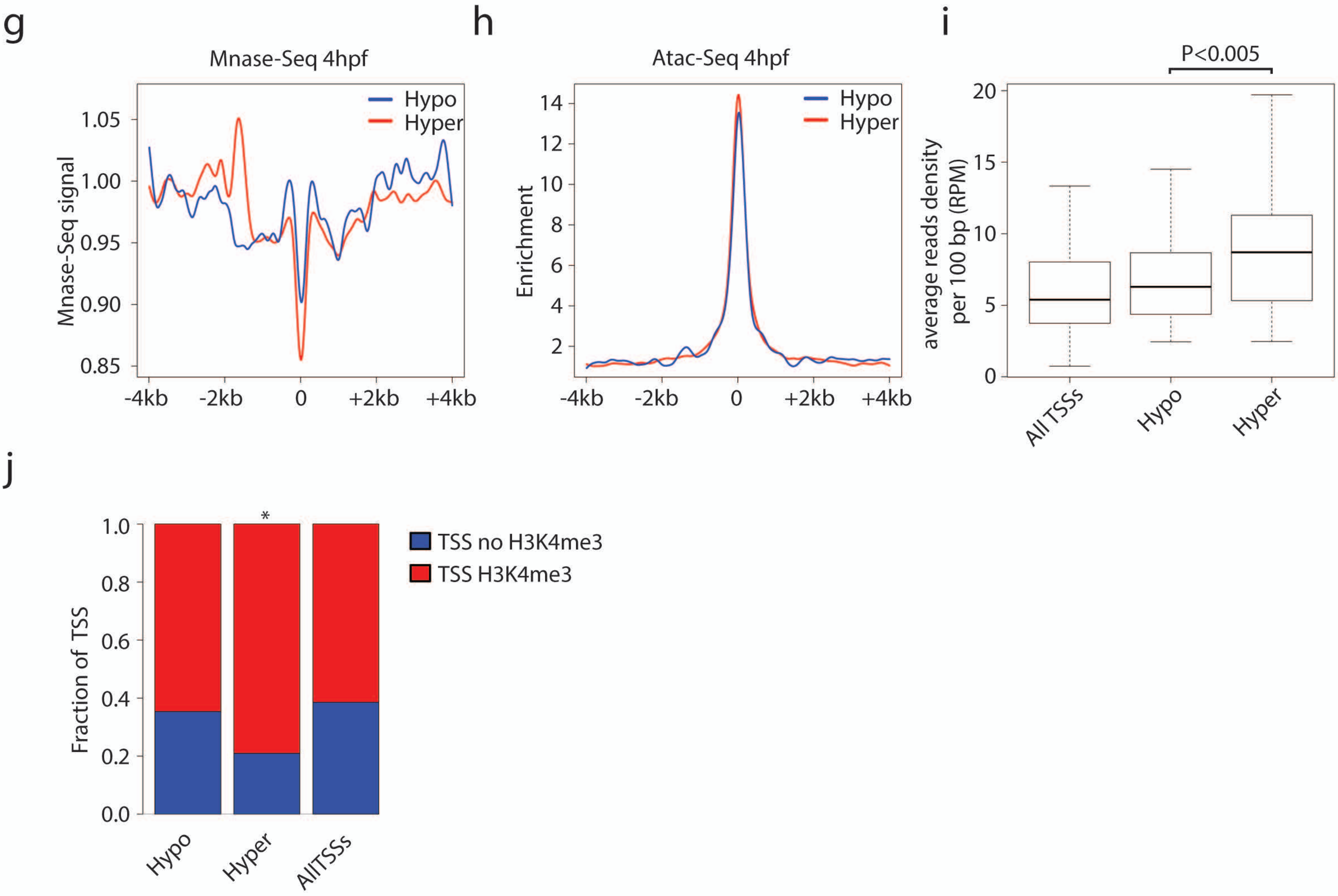
g
Mnase-Seq 4hpf
Hypo
Hyper
Mnase-Seq signal
1.05
1.00
0.95
0.90
0.85
-4kb
-2kb
0
+2kb
+4kb
h
Atac-Seq 4hpf
Hypo
Hyper
Enrichment
14
12
10
8
6
4
2
-4kb
-2kb
0
+2kb
+4kb
i
P<0.005
average reads density
per 100 bp (RPM)
20
15
10
5
0
All TSSs
Hypo
Hyper
j
Fraction of TSS
1.0
0.8
0.6
0.4
0.2
0.0
*
TSS no H3K4me3
TSS H3K4me3
Hypo
Hyper
AllTSSs

# Figure S4

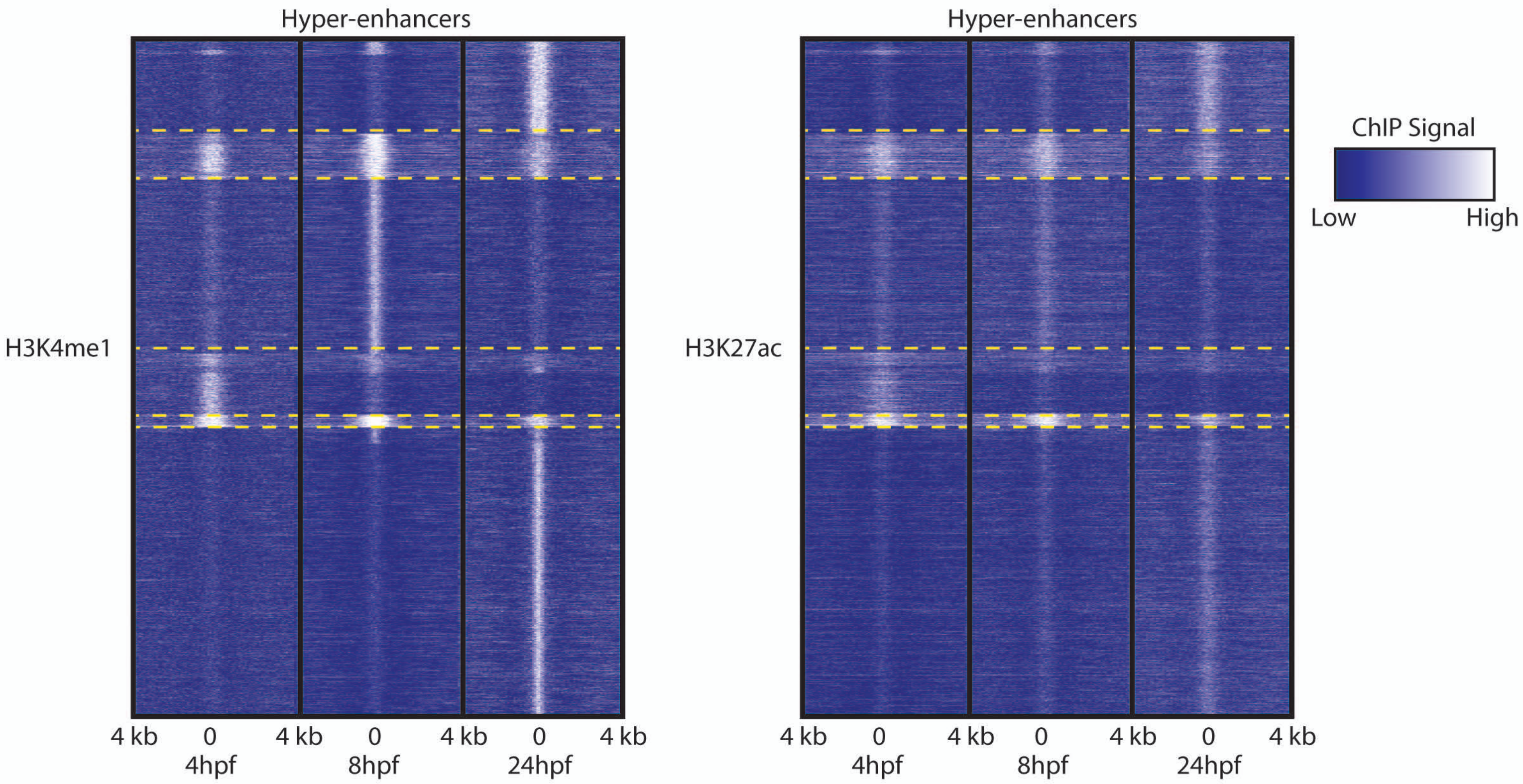

Hyper-enhancers
Hyper-enhancers
H3K4me1
H3K27ac
ChIP Signal
Low
High
4 kb
0
4 kb
0
4 kb
0
4 kb
4hpf
8hpf
24hpf
4 kb
0
4 kb
0
4 kb
0
4 kb
4hpf
8hpf
24hpf

Figure S5

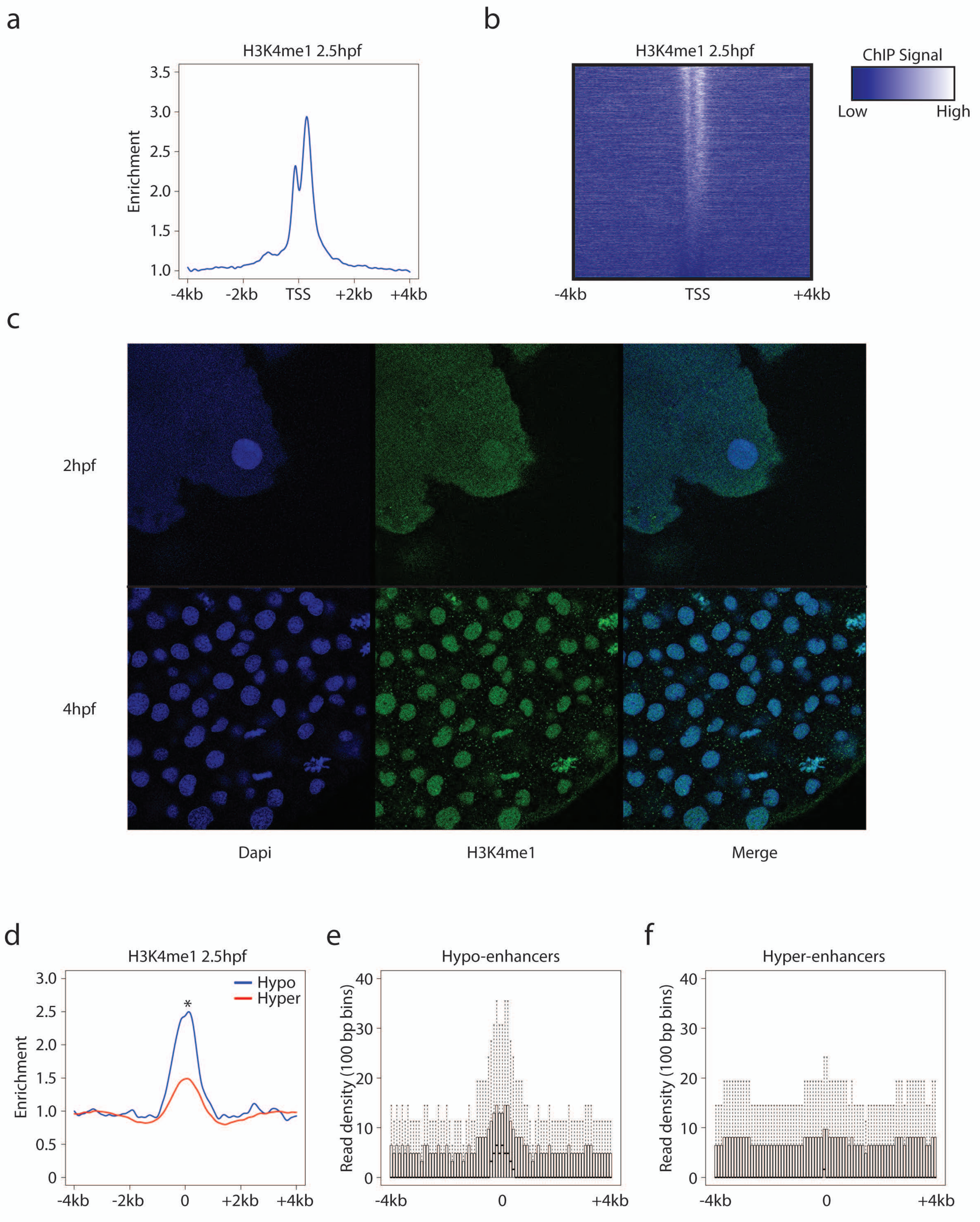
a
H3K4me1 2.5hpf
Enrichment
3.5
3.0
2.5
2.0
1.5
1.0
-4kb
-2kb
TSS
+2kb
+4kb
b
H3K4me1 2.5hpf
ChIP Signal
Low
High
-4kb
TSS
+4kb
c
2hpf
4hpf
Dapi
H3K4me1
Merge
d
H3K4me1 2.5hpf
Hypo
Hyper
*
Enrichment
3.0
2.5
2.0
1.5
1.0
0.5
0
-4kb
-2kb
0
+2kb
+4kb
e
Hypo-enhancers
Read density (100 bp bins)
40
30
20
10
0
-4kb
0
+4kb
f
Hyper-enhancers
Read density (100 bp bins)
40
30
20
10
0
-4kb
0
+4kb

# Figure S6

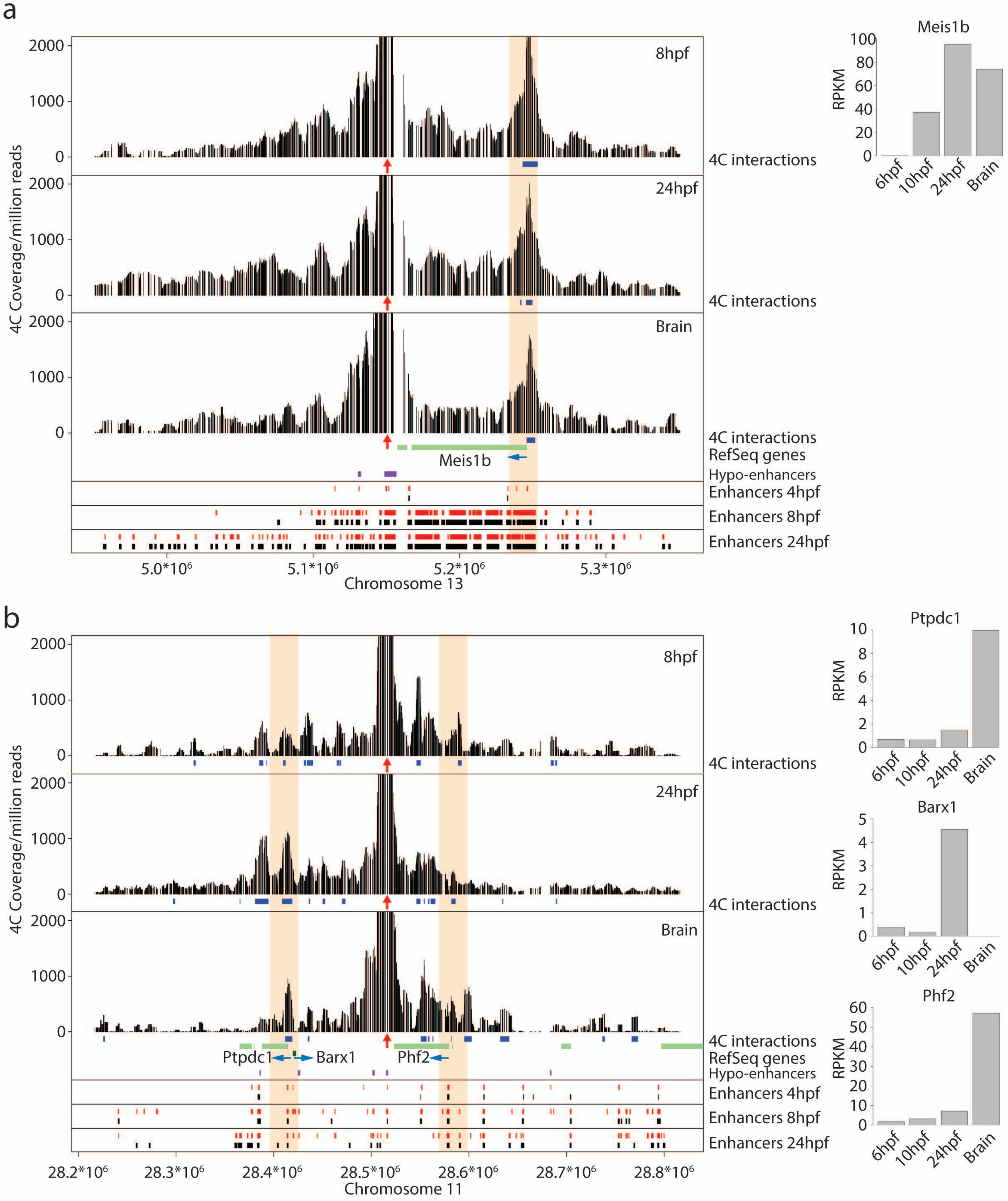
a
4C Coverage/million reads
2000
1000
0
8hpf
24hpf
Brain
4C interactions
RefSeq genes
Meis1b
Hypo-enhancers
Enhancers 4hpf
Enhancers 8hpf
Enhancers 24hpf
5.0*10⁶
5.1*10⁶
5.2*10⁶
5.3*10⁶
Chromosome 13
Meis1b
RPKM
100
80
60
40
20
0
6hpf
10hpf
24hpf
Brain
b
Ptpdc1
Barx1
Phf2
28.2*10⁶
28.3*10⁶
28.4*10⁶
28.5*10⁶
28.6*10⁶
28.7*10⁶
28.8*10⁶
Chromosome 11
10
8
6
4
2
5
3
1
60
50
40
30
10

# Figure S 6

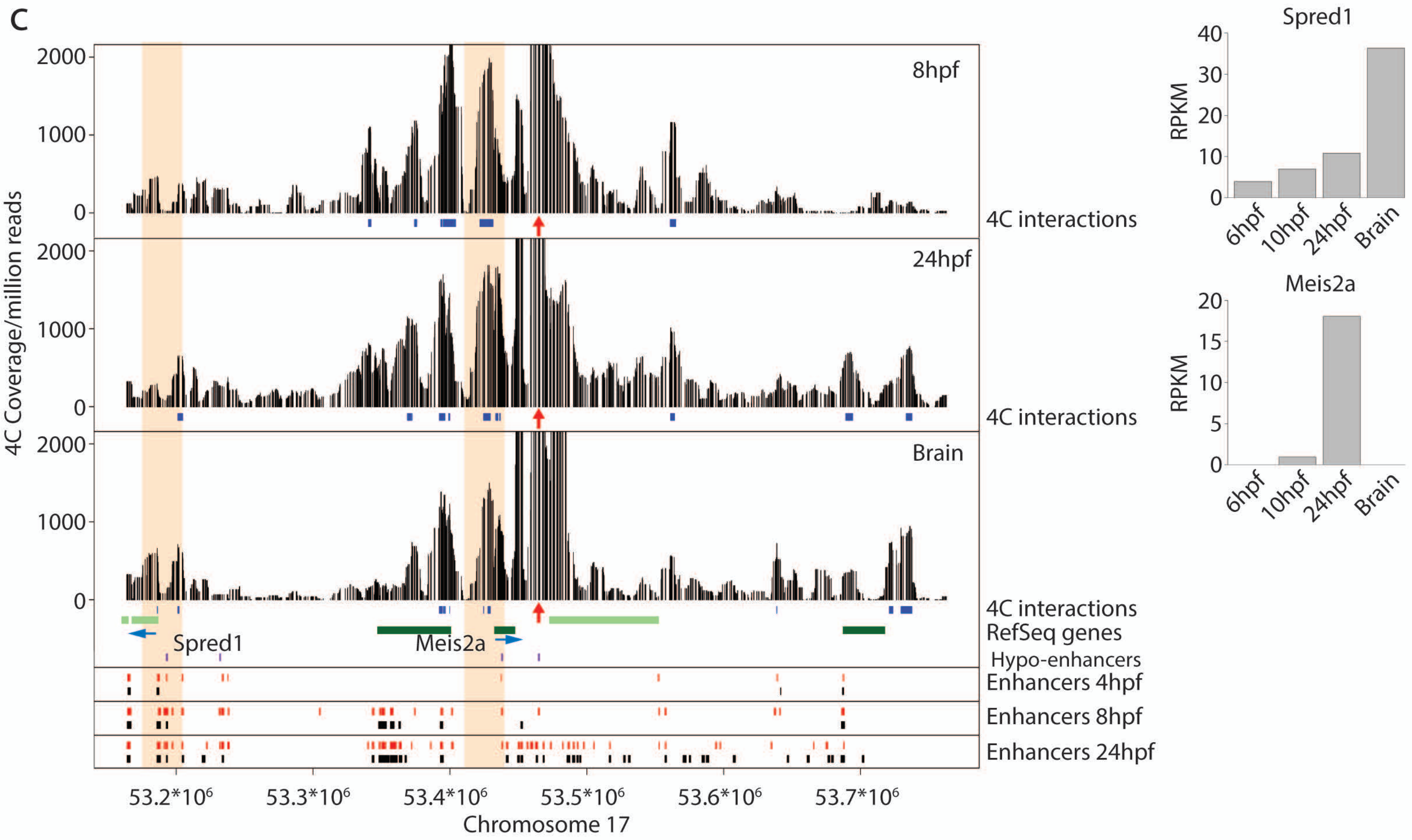
c
8hpf
24hpf
Brain
4C Coverage/million reads
2000
1000
0
4C interactions
RefSeq genes
Spred1
Meis2a
Hypo-enhancers
Enhancers 4hpf
Enhancers 8hpf
Enhancers 24hpf
53.2*10⁶
53.3*10⁶
53.4*10⁶
53.5*10⁶
53.6*10⁶
53.7*10⁶
Chromosome 17
Spred1
RPKM
40
30
20
10
0
6hpf
10hpf
24hpf
Brain
Meis2a
RPKM
20
15
10
5
0
6hpf
10hpf
24hpf
Brain
