# Supplementary material for: Enhancers reside in a unique epigenetic environment during early zebrafish development
Source: Genome Biol. 2016 Jul 5;17:146. doi: 10.1186/s13059-016-1013-1 (PMC4934011; doi:10.1186/s13059-016-1013-1)
Supplement: Additional file 1: — Figures S1–S6. (ZIP 3593 kb) [file 13059_2016_1013_MOESM1_ESM.zip › Supplemental Information.pdf]

Supplemental Information to:

Enhancers reside in a unique epigenetic environment during early development.

Lucas J. T. Kaaij[1], Michal Mokry[2, 6], Meng Zhou[3, 6], Michael Musheev[1], Geert Geeven[4], Adrien S. J. Melquiond[4], António M. de Jesus Domingues[1,] Wouter de Laat[4], Christof Niehrs[1, 5], Andrew D. Smith[3], René F. Ketting[1*].

1 Institute of Molecular Biology (IMB), Ackermannweg 4, D-55128 Mainz, Germany.

2 Department of Pediatric Gastroenterology, Wilhelmina Children's Hospital, University Medical Centre Utrecht, 3508 AB Utrecht, the Netherlands

3 Molecular and Computational Biology, University of Southern California, Los Angeles, CA 90089, USA

4 Hubrecht Institute-KNAW & University Medical Centre Utrecht, Uppsalalaan 8, 3584 CT Utrecht, The Netherlands

5 Division of Molecular Embryology, DKFZ-ZMBH Alliance, D-69120 Heidelberg, Germany

6 Equal contributions

*Correspondence: r.ketting@imb.de

Figure S1. Epigenetic interactions at all TSSs and lack of DNA methylation dynamics at enhancers during early development.

(a) Heatmap displaying epigenetic environment of all refseq TSS at 4hpf. The heatmap was sorted on H3K4me1 peak intensity. (b) Violin plots displaying the DNA methylation state of 4hpf enhancers in zebrafish and enhancers called in mESC and their DNA methylation state in mESC, ICM and e6.5 embryos, as indicated. (c) Violin plots displaying the DNA methylation status of exons and introns in zebrafish and mESC, as indicated. (d) Violin plots displaying the DNA methylation status of hyper-enhancers that are shared between 4 and 8hpf in adult muscle as indicated. (e) Upper panel. Heatmaps displaying normalized H3K27ac peak intensities uniquely present at one timepoint. Lower panel: Displays the average DNA methylation observed in the different methylomes at the underlying DNA of active enhancers as separated in the upper panel.

FigureS2. Hypo-enhancers show reproducible expression pattern in enhancer assays. (a,b) Genome browser views of the two hypo-enhancers used in panel c. ChIP-Seq data and DNA methylation data are displayed as indicated from 4hpf old embryos. Normalized ChIP-Seq enrichments and fractional methylation are indicated at the left site of the image. Blue box indicates the hypo-enhancer. The blue arrow indicates the orientation of the gene. Blue horizontal bars indicate HMRs. (c) *in vivo* enhancer assay of hypo-enhancers located within 20kb of the indicated genes (see genome browser view S2a,b). The images were taken 24hpf and 48hpf of *tbx2a* and *dachA* respectively. (d,e) Genome browser view of the two hypo-enhancers tested in f and g. ChIP-Seq data and DNA methylation data are displayed as indicated from 4hpf old embryos.

Normalized ChIP-Seq enrichments and fractional methylation are indicated at the left site of the image. Blue box indicates the hypo-enhancer. The blue arrow indicates the orientation of the gene. Blue horizontal bars indicate HMRs. (f,g) *in vivo* enhancer assay of hypo-enhancers located within 20kb of the indicated genes. The images were taken 3dpf and 10hpf of *gsc* and *unxc4.1*, respectively.

FigureS3. Confirmation of the quality of the Atac-Seq data and the epigenetic environment of TSS close to hypo- and hyper-enhancers.

(a) Composite plot displaying in the upper and middle panel Mnase-Seq and Atac-Seq read density over all refseq TSSs, respectively. The heatmap (lower panel) shows normalized read density over all refseq TSSs of the Atac-Seq dataset. (b) Profiles of sheared DNA obtained from 4hpf old embryos. The enrichment (Y-axis) of the sheared DNA is plotted over a region of 4kb up- and down-stream of the middle points of the two-enhancer types.

(c-f) Boxplot representation of the H3K4me3 and H3K4me2 read density over hypo- and hyper-enhancers, as indicated. (g,h) Profiles displaying normalized read density of Mnase-Seq and Atac-Seq over TSSs within 50kb of a hypo- or hyper-enhancers as indicated (i) Boxplot representing PolII read density at TSS (plus and minus 500 bp) at all TSSs or TSSs within 50kb of a hypo- or hyper-enhancers as indicated. (j) Barplot showing the overlap of all TSSs or TSSs within 20kb of a hypo- or hyper-enhancers with H3K4me3 as indicated (*=p-value <0.001, hyper geometric distribution).

Figure S4. The presence of stage specific H3K4me1 coincides with stage specific H3K27ac

Heat-map displaying normalized H3K27ac and H3K4me1 intensities of all hyper-enhancers called throughout the first 24 hours of development as indicated. The heat-map was clustered using the same parameters as in Figure 4a. Yellow dashed lines indicate cluster transitions.

Figure S5. H3K4me1 is enriched at TSSs and hypo-enhancers at 2.5hpf

(a) Profiles of H3K4me1 ChIP-Seq at 2.5hpf over all refseq TSSs (b) Similar as in (a), but now represented as a heatmap. (c) Immuno staining against H3K4me1 at 2hpf and 4hpf. (d) Profiles of H3K4me1 ChIP-Seq at 2.5hpf over hypo- and hyper-enhancers called at 4hpf. (e,f) Similar as in (d), but now visualized in a boxplot in order to show the various enhancers individually.

Figure S6. 4C-Seq reveals that hypo-enhancers interact throughout development with its target TF

(a-c) 4C-Seq profiles generated at 8hpf, 24hpf and from brain as indicated. Red vertical arrows under every 4C-Seq panel indicate the viewpoint. All 4C interactions are indicated by blue rectangles below every 4C-Seq panel All significant 4C interactions are indicated by blue rectangles below every 4C-Seq panel and 4C interactions with a TSS are highlighted in orange. Below the 4C-Seq panels tracks indicating hypo-enhancers (purple) and ChIP-Seq peaks (H3K4me1 (red) and H3K27ac (black)) throughout development are shown. The expression of the gene(s) with a 4C interaction at the TSS is displayed on the right of the 4C-Seq panels.
